# Upcycling agro-industrial blueberry waste into platform chemicals and structured materials for application in marine environments†

**DOI:** 10.1039/d2gc00573e

**Published:** 2022-04-07

**Authors:** Guillermo Reyes, Claudia M. Pacheco, Estefania Isaza-Ferro, Amaidy González, Eva Pasquier, Serguei Alejandro-Martín, Luis E. Arteaga-Peréz, Romina R. Carrillo, Isabel Carrillo-Varela, Regis Teixeira Mendonça, Colleen Flanigan, Orlando J. Rojas

**Affiliations:** Biobased Colloids and Materials, Department of Bioproducts and Biosystems, School of Chemical Engineering, Aalto University FI-00076 Espoo Finland guillermo.reyes@aalto.fi orlando.rojas@ubc.ca; Facultad de Ingenierías, Universidad Cooperativa de Colombia Cra 22 No. 7-06 sur Villavicencio Colombia; Department of Bioproducts and Biosystems, School of Chemical Engineering, Aalto University FI-00076 Espoo Finland; Laboratory of Thermal and Catalytic Processes, Facultad de Ingeniería, Universidad del Bío-Bío Av. Collao 1202 Concepción Chile; Université Grenoble Alpes, CNRS, Grenoble INP (Institute of Engineering) LGP2 F-38000 Grenoble France; Laboratorio de Cromatografía Gaseosa y Pirólisis Analítica, Departamento de Ingeniería en Maderas, Universidad del Bío-Bío Av.Collao 1202, Casilla 5-C Concepción Chile; Facultad de Ciencias Químicas, Depto. Química Analítica e Inorgánica, Universidad de Concepción Concepción Chile; Laboratorio de Recursos Renovables, Centro de Biotecnología, Universidad de Concepción, Concepción Casilla 160-C Concepción Chile; Centro de Investigación de Polímeros Avanzados, CIPA, Avenida Collao 1202, Edificio de Laboratorios Concepción 4030000 Chile; Facultad de Ciencias Forestales, Universidad de Concepción Casilla 160-C Concepción Chile; Zoe – A Living Sea Sculpture in Cozumel, Av. Rafael E. Melgar 77688 San Miguel de Cozumel Q.R. Mexico; Bioproducts Institute, Department of Chemical & Biological Engineering, Department of Chemistry and Department of Wood Science, 2360 East Mall, The University of British Columbia Vancouver BC V6T 1Z3 Canada

## Abstract

Blueberry pruning waste (BPw), sourced as residues from agroforestry operations in Chile, was used to produce added-value products, including platform chemicals and materials. BPw fractionation was implemented using biobased solvents (γ-valerolactone, GVL) and pyrolysis (500 °C), yielding solid fractions that are rich in phenols and antioxidants. The liquid fraction was found to be enriched in sugars, acids, and amides. Alongside, filaments and 3D-printed meshes were produced *via* wet spinning and Direct-Ink-Writing (DIW), respectively. For the latter purpose, BPw was dissolved in an ionic liquid, 1-ethyl-3-methylimidazolium acetate ([emim][OAc]), and regenerated into lignocellulose filaments with highly aligned nanofibrils (wide-angle X-ray scattering) that simultaneously showed extensibility (wet strain as high as 39%). BPw-derived lignocellulose filaments showed a tenacity (up to 2.3 cN dtex^−1^) that is comparable to that of rayon fibers and showed low light reflectance (*R*_ES_ factor <3%). Meanwhile, DIW of the respective gels led to meshes with up to 60% wet stretchability. The LCF and meshes were demonstrated to have reliable performance in marine environments. As a demonstration, we show the prospects of replacing plastic cords and other materials used to restore coral reefs on the coast of Mexico.

## Abstract 2

Agriculture residues from blueberries were transformed into added-value products and materials using γ-valerolactone and an ionic liquid.

## Graphical abstract

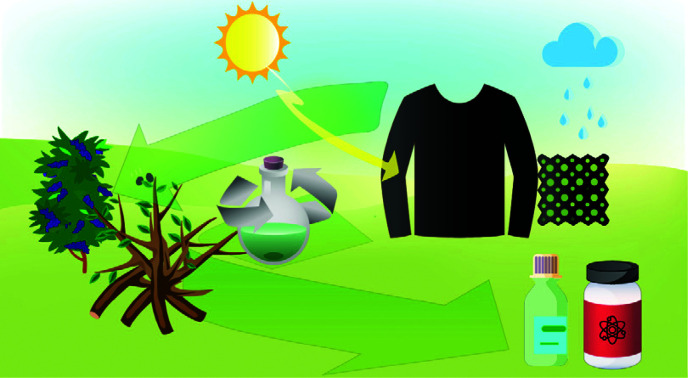

## 1. Introduction

Modern industry is focusing its attention on the use of sustainable resources.^1^ Due to their intrinsic properties, lignocelluloses from forest and agriculture residues are promising feedstocks for biorefinery and material production.^2^ The use of agricultural biomass sources to produce biofuels and bioproducts from biorefineries represents a foundational approach for the synergistic coproduction of power, heat, and biofuels alongside materials in short-cyclic CO_2_ processes.^3^ Of relevance to this discussion is the blueberry pruning waste (BPw) suggested as a suitable bioresource given its rich chemical composition and wide availability.^4–6^ BPw is produced in the pruning stages of blueberries, at a rate of *ca.* 3000–7500 kg per planted hectare.^7^ Considering the annual blueberry world production (*ca*. 820 thousand metric tonnes)^8^ and composition,^9^*ca*. 180–460 thousand metric tonnes of cellulose and 100–190 thousand metric tonnes of lignin are estimated to be available for processing.

In this context, biorefineries based on agroforestry resources can be considered for energy, high added-value chemicals, and material markets,^1,2^ enabling a paradigm shift towards the material and energy use within a closed-loop concept in a cradle-to-cradle approach.^10^ However, any success depends on applying integrated approaches that take advantage of the chemical diversity, which can use solvolysis-based strategies integrated with thermal or catalytic processing.

Biobased solvents such as γ-valerolactone (GVL) have found application in the primary processing of lignocellulose and the extraction of multiple added-value products.^11–14^ Similarly, some ionic liquids are promising green solvents given their high thermal stability, no volatility, and effectiveness in the selective dissolution of lignocellulose products.^15^ Different IL solutions that dissolve cellulose without a significant decrease in the polymerization degree have been demonstrated.^16^ The limited depolymerization after dissolution allows a platform for obtaining specialty chemicals (*i.e.*, furanics) or backbone components for functional materials. Ionic liquids, such as 1-ethyl-3-methylimidazolium hydrogen sulfate [emim][HSO_4_] and [emim][OAc], have shown an excellent performance in the fractionation of wheat straw, for instance, producing phenols^17,18^ and glucose^18^ in high yields. In particular, [emim][OAc] exhibited tunable acid/base behavior to dissolve, fractionate and functionalize cellulose and lignin,^19,20^ suggesting the potential to produce functional materials. This possibility has been recently demonstrated in filaments obtained from recycled pulps or recovered textiles using Protic Ionic Liquids (PILs),^21^ exemplifying a disruption of traditional processing and as an option to reduce CO_2_ emissions and microplastic generation.^22,23^ Nevertheless, several challenges exist in the IL biomass-to-textile production chain; for instance, IL-based technologies should include a strategy for an integrated valorization of the liquid and solid fractions. Therefore, alternative processes should be assessed for maximizing profitability under minimum or controlled environmental impacts.

Our study proposes an integrated and scalable approach for waste upcycling into high-value products (bioproducts) and high-volume platform chemicals. Our approach for BPw feedstock processing uses green solvents (GVL, IL) to simultaneously produce platform chemicals and filaments. The process flexibility enhances the techno-economic viability and considers the demands at different levels of the value chain.^24,25^ Related approaches have been proposed to valorize agro-industrial residues such as corn stover,^26^ sugarcane,^27^ marine,^25^ and farming^28^ waste. The valorization strategy considers two alternatives: GVL solvolysis in the first conversion stage, followed by the thermal valorization of solids *via* fast pyrolysis at 500 °C to produce different liquid fractions rich in sugars, phenols, and furanics. Another stage uses dissolution in the commercial IL [emim][OAc], allowing the production of lignocelluloses suitable for the production of filaments (LCF) and meshes.

## 2. Materials and methods

**Fig. 1:**
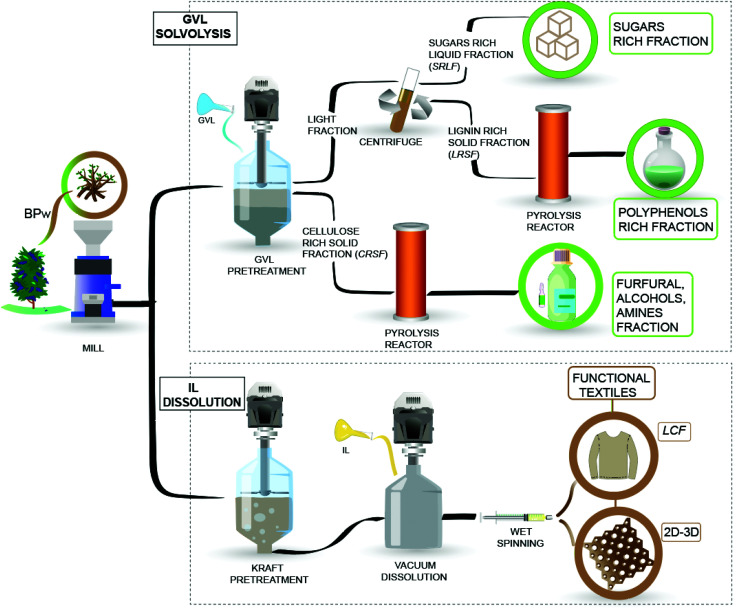
Integrated approach for upcycling BPw into added-value chemicals and functional filaments.

BPw with a known chemical composition^9^ was milled (45–60 mesh) and dried (101 °C, 12 h) to produce chemicals and functional filaments, following an integrated process, Fig. 1. In addition to the solvolysis processes, pyrolysis has been shown to be an effective way to convert biomass into a liquid (bio-oil) mixture of chemicals.^29,30^ However, the low yields of chemicals and the high oxygen content of bio-oils limit the application of pyrolysis on a large scale.^30^ In this sense, the integration of pyrolysis with solvolytic depolymerization is a plausible way to produce bio-oils as a platform for chemical or material synthesis. Fig. 1 shows the proposed technology considering GVL and IL solvolysis and dissolution, respectively. The process is divided into two stages, described as follows.

### 2.1 GVL solvolysis and pyrolysis

The solvolysis was performed in a single step in a Parr 4564 reactor (Parr® Instrument Company, Illinois, USA), mixing (300 rpm) 1 g of BPw (ground and sieved) into 10 mL solution (GVL/H_2_O, 50% V/V) at (70 °C, 6 h,), following a procedure reported elsewhere.^13^ At the end of the reaction, the mixture was filtered, obtaining a Cellulose Rich Solid Fraction (CRSF) and a light fraction, Fig. 1. The light fraction was consecutively centrifuged at 6000 rpm for 10 minutes (Centurion Scientific K2015, UK) to isolate a Lignin Rich Solid Fraction (LRSF) and Sugar Rich Liquid Fraction (SRLF). The latter fraction was analyzed by High-Performance Liquid Chromatography (HPLC) and derivatized liquid fraction analysis by Gas Chromatography-Mass Spectrometry (der-GC/MS)^31^ (ESI, protocols 2.1 and 2.2†). Meanwhile, the solid fractions (CRSF and LRSF) were characterized by Fourier-transform infrared spectroscopy with an attenuated total reflectance accessory (FTIR-ATR) and used as feedstocks for pyrolysis in a micro-pyrolysis unit (CDS Analytical Pyroprobe (5200 HPR), coupled to a Gas Chromatograph (Clarus 690, PerkinElmer) equipped with a mass spectrometer (Clarus SQ8 T) (ESI, protocol 2.3†).

### 2.2 IL dissolution towards functional textiles

The BPw material previously dried and milled underwent alkaline Kraft pre-treatment with a variable active alkali content (expressed as sulfidity), namely 22%, 24%, and 26% to produce three samples, namely *A*, *B*, and *C*, respectively. We noted that the spent liquor can be subjected to a well-established recovery cycle that leads to the (a) concentration and recovery of digestion chemicals; for instance, inorganic sodium salts which are converted into white liquor, and (b) combustion of the organic fraction (mostly lignin) for energy co-generation.^32^ Related efforts also consider the valorization of tall oils and associated fractions, which can be useful for biobased polyol and phenol syntheses as well as lignins (in boiler-limited operation) that can be converted into particles,^33^ or used in coatings, paints, drying oils, emulsions, and lubricants.^34^

The samples (*A*, *B*, and *C*) corresponding to 5.5, 4, and 3% lignin contents were dissolved using the IL [emim][OAc] (CAS No. 143314-17-4, purity = 97%, Sigma Aldrich) to produce lignocellulose filaments or (LCF) and printed meshes. The dissolution process started with drying the samples at 60 °C, 200 mbar, 16 h. The samples (*A, B*, and *C*) were used to prepare dissolved dopes at 6% w/w cellulose content. Sample *D* was obtained from sample *A* at a half cellulose concentration (3% w/w) under the same dissolving conditions. The Pulp Yield, Kappa Number (KN), viscosity, fiber length, and fiber width were assessed (ESI, protocol 2.4†). The rheology behavior of dissolved dopes was assessed (ESI,† method 3.1), and then the dopes were used to produce different filaments (LCF) and 3D printed materials (ESI,† method 3.2). The mechanical properties of these regenerated materials were assessed by tensile tests (Young's Modulus, tensile strength, toughness, tenacity, shrinkage, and swelling). LCF sample's orientation, crystallinity index, and lateral crystallite size (2 0 0) were obtained from bench beamline SAXS/WASX unit measurements. The thermal stability was studied using a thermogravimetric analyzer (ESI,† method 3.3). The spun and printed materials were also characterized under wet conditions (swelling, shrinkage, stretching) (eqn (S2)–(S4)†) for evaluation in the ocean environment.

## 3. Results and discussion

### 3.1 Fractionation with GVL and pyrolysis

**Fig. 2:**
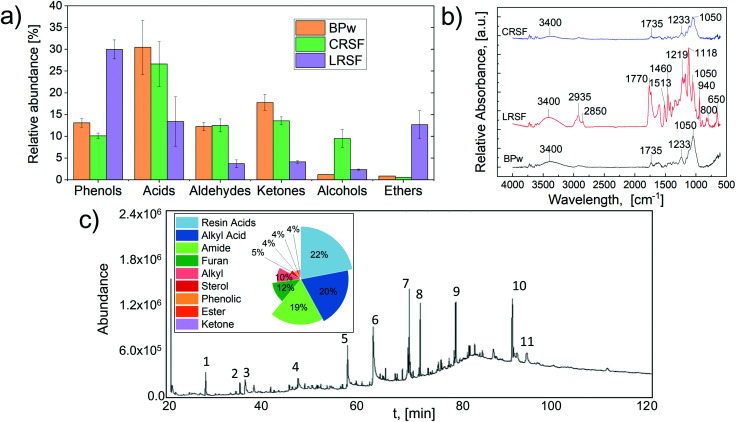
Chemical composition before and after GVL solvolysis of solid fractions (CRSF, LRSF) and a liquid fraction (SRLF): (a) solid fractions’ pyrolytic gaseous stream chemical composition. (b) solid fractions’ FTIR spectra. (c) Liquid fraction (SRLF) derivatized and analyzed by Gas Chromatography-Mass Spectrometry (der-GC/MS).

Fig. 2 shows the chemical composition of the different fractions using different instrumental techniques.

The proper selection of fractionation and pre-treatment chemicals is a critical factor for product isolation from thermochemical processes.^35^ The use of solvolysis combined with pyrolysis is one of the preferred technologies to convert lignocellulose biomass into high added value streams.^2^ Herein, the chemical composition of the vapors obtained from the pyrolysis of CRSF and LRSF streams was investigated by Py-GC/MS. Fig. 2a shows the area-related selectivity of the significant chemical families detected by GC–MS analysis of pyrolysis vapors.

Additionally, the FTIR-ATR spectra of solid fractions, before and after solvolysis treatment with GVL, are presented in Fig. 2b. The liquid fraction (SRLF) was first analyzed by HPLC-UV/RID and UHPLC-MS/MS to detect high molecular weight compounds. In contrast, the family of low molecular weight compounds was analyzed by derivatization and Gas Chromatography-Mass Spectrometry (der-GC/MS) (Fig. 2c). The experimental results in Fig. 2 confirmed that the chemical pre-treatment before pyrolysis led to a more uniform bio-oil composition. For instance, the pyrolysis of the CRSF led to the formation of amines and alcohols. Meanwhile, the formation of phenols, aromatics, and ethers was favored during LRSF pyrolysis, which is in line with the aromatic nature of lignin, as previously demonstrated.^36^ The formation of ketones and acids during the pyrolysis of BPw and solid fractions from solvolysis demonstrated a reduction for CRSF and LRSF compared to the untreated BPw. This result is highly relevant since bio-oils with low acid content are relatively stable and can be used as fuels without further treatment.^37^ A detailed report on the chemical composition of the solid fraction (BPw, CRSF, and LRSF) vapors from pyrolysis is available as supplementary data (Table S1†). The primary compounds for each fraction and their relative abundance (eqn (S1)†) were as follows: CRSF: 27% acids (15.5% acetic acid), 14% ketones (3.2% 1-hydroxy-2-propanone), and 13*%* aldehydes (4.4% methylglyoxal, 3.9% furfural, and 1.8% hydroxy-acetaldehyde). LRSF indicated a high fraction of polyphenols 30% (7.7% 2,6-dimethoxy-phenol and 5.5% 2-methoxy-4-vinyl phenol) and ethers 13% (10% furans). Among the obtained species, 1-hydroxy-2-propanone, also known as acetol, can be used as an organic intermediate (it contains both hydroxyl and carbonyl functional groups) to enhance reactions (dehydration, hydrogenation, and oxidation) to produce propylene glycol, acrolein, acetone, and furan derivatives.^38^ In the same way, some of the obtained products can be valorized and used for other purposes; *e.g.*, hydroxy-acetaldehyde, an effective meat-browning agent, and a fermentation feedstock for ethylene glycol production; acetic acid, for cellulose acetate and polyvinyl acetate production.^39^ Furfural can be converted to 2-methylfuran over supported biobased catalysts.^40^ Furthermore, high phenolic concentrations on fractionated lignocellulose streams have been shown to boost antioxidant activity.^41^

The pretreatment of lignocellulose samples using GVL-based solvolysis demonstrates an effective pathway to obtain platform chemicals and alternative quality biofuels that can be recovered by a combined cascading approach.^41^ Further analysis by FTIR shown in Fig. 2b demonstrates that the IR bands of the LRSF in the fingerprint region (1770–800 cm^−1^) are considerably different from those of BPw and CRSF. These compositional differences can explain the results obtained from pyrolysis and demonstrate the effectivity of the GVL-solvolytic process to depolymerize BPw. In fact, the complex IR absorbance signals of the LRSF indicate that this solid fraction might be rich in methoxyl–O–CH_3_, C–O–C stretching, and C=C stretching (aromatic ring) containing compounds.

The FTIR spectra of BPw indicate a typical lignocellulosic biomass profile showing characteristic peaks at 1735 cm^−1^ ascribed to the C=O stretching. In contrast, the signals at 1232 cm^−1^ and 1050 cm^−1^ represent the aryl-alkyl ether linkages and C–O stretching, respectively. The broad signal found in the three solid samples at 3400 cm^−1^ was ascribed to the –OH stretching. The spectra of the CRSF were similar to those of BPw but with a reduction in the C=O stretching (1735 cm^−1^), which disappeared for LRSF. This observation for the carboxyl-related signal correlates with the content of acids, aldehydes, and ketones in the pyrolysis vapors, following the ranking BPw > CRSF > LRSF. In addition, the C–H_*n*_ stretching for aromatics and the C=O stretching for aromatic skeletal stretching (2850–2935 cm^−1^, 1770 cm^−1^, and 1610 cm^−1^) were only detectable for LRSF, as expected and in line with vapor composition.

The liquid fraction extracted with GVL (SRLF) was analyzed by HPLC-UV/RID and revealed a complex mixture of organic acids and carbohydrates.^42^ Among these, fructose, formic and acetic acids were identified by matching their retention time with the standards (Fig. S1†). Furthermore, other unknown compounds with high activity in the refractometric index (RID) suggested the presence of high molecular weight (MW) carbohydrates. UHPLC-MS/MS assays were performed to analyze unknown signals and the signals corresponded to high MW organic acids, lignan glycosides, and other higher MW derivatives in the GVL extract (Fig. S2†). On the other hand, following GC/MS analyses, the derivatized samples of the GVL extract showed numerous signals (Fig. 2c), corroborating that the derivatization process, using BSTFA + TMCS, improved the volatility of many of the components. These signals covered almost 85% of the total area of the chromatogram, corresponding to the volatile components found in a more significant proportion in the sample (Table S2†). Among the components with the highest abundance (area), it was possible to identify peak 6 (20.5%, Fig. 2c), corresponding to *cis-vaccenic acid*, an isomer of *oleic acid*. This product is essential in the food industry, with multiple dairy product applications.^43^ On the other hand, peak 10 (14.9%), attributed to *taraxerone*, a pentacyclo-triterpenoid, is widely reported for its immune-modulating effect, as well as antifungal and antiviral activities.^44^ In addition, other components found are important chemical platforms, such as 5-HMF (peak 3), widely used in the production of inks, dyes, personal care products, rubber, and plastic, among others.^45^ The main compounds were grouped according to their main functional groups. In the extract, the more abundant fractions corresponded to resin acids (22%), alkyl acids (20%), and amides (19%). Present in lower percentages, we found furans (12%), hydrocarbons (alkyl, 10%), and sterols (5%), among others. The low carbohydrate content detected by der-GC/MS was explained by the fact that this technique does not have the same sensitivity as HPLC for highly polar compounds with low volatility. HPLC analyses indicated a high content of sugars, mainly in the form of lignan glycosides, which have been widely studied in recent years for their reported health benefits.^46^ The purification and isolation of individual chemicals from BPw *via* GVL fractionation and pyrolysis is a significant challenge. Drugkar *et al.*^47^ recently discussed separation technologies for upgrading bio-oils and proposed the separation of bio-oils into fractions with more defined chemical profiles, still mixtures but more amenable for further fractionation and chemical recovery. Fractionation and solvent extraction are the most promising technologies for isolating specific chemicals or producing narrower bio-oil functional group profiles. In the case of the polyphenol-rich fraction, organic solvents such as hexane, ethyl acetate, petroleum ether, and chloroform show great potential for the recovery of chemicals.^48,49^ On the other hand, HMF and furanic fractions can be extracted with chloroform to separate purer species.^47^

### 3.2 Valorization by ionic liquid dissolution

Following the proposed fractionation process towards functional materials, BPw was pretreated by an alkali kraft procedure. The alkali charge effects on lignin percentage, viscosity, and fiber dimensions are shown in Table S3.† The results demonstrated that a high alkali content facilitated lignin removal; however, the pulp yield remained relatively stable (35%): the alkali charge did not produce significant changes in cellulose accessibility. By increasing the alkali charge from 22 to 26%, both the lignin content and pulp viscosity decreased: from 5.6% to 3% for lignin and from 1081 to 980 ml g^−1^ for viscosity. This effect directly influences the processability of pulps as raw materials in producing materials and their mechanical properties. The removal of lignin and hemicelluloses reduced the viscosity; therefore, a low alkali charge effectively preserved cellulose chains, providing fibers with a greater length and width. The length of BPw fibers (0.5 mm) was shorter compared to that of other agroforestry residues, such as cotton stalks (0.81 mm), wheat straw (0.74 mm), rice straw (0.89 mm), and canola stalks (1.17 mm).^50^ Furthermore, the increase in alkali enhanced the brightness of the pulp, related to the decrease in lignin content.^51^ The different pretreated pulps (*A*, *B*, and *C*) with lignin percentages of 5.6, 4.1, and 3% were used as raw materials to produce different materials, as described in the following sections.

#### LCF filaments

**Fig. 3:**
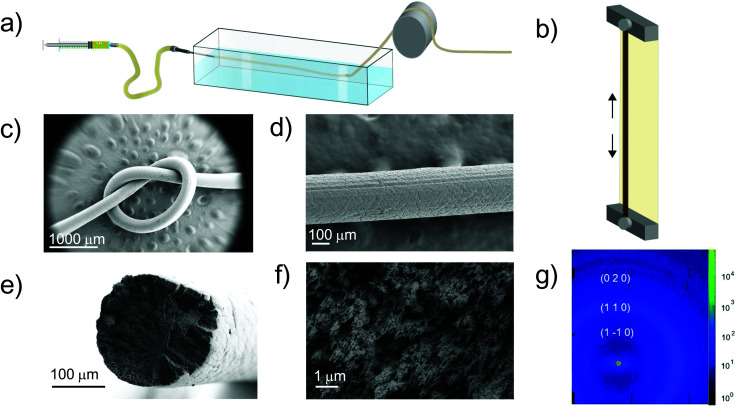
Wet spinning process and LCF morphology: (a) wet spinning setup. (b) Filament drying under tension. (c) SEM image of a filament knot. (d) LCF surface texture and (e) cross-section in low and (f) high magnification. (g) LCF WAXS azimuthal peak intensity distribution.

The wet-spinning process was used to produce LCF, see Fig. 3 and supporting video **Spinning_LCF.mp4**.† After extrusion in the coagulation bath, the regeneration process involved IL solvent exchange with water. The IL can be optionally separated by distillation or ionic exchange adsorption technologies, as shown in earlier reports.^52^ The regenerated LCF was collected in a rotating drum, cut into small pieces (0.5 m length), and dried under tension (Fig. 3a and b). Cellulose-based filaments were dried under tension (by holding the filaments on each end) to produce elongational stress forces resisting shrinkage, favoring alignment, and potentially improving the filaments’ toughness.^53^

The rheology and flowability of the precursor solution were crucial parameters to reach optimal process conditions for the extrusion of the lignocellulose materials where relatively low viscosities and liquid-like behavior are often desirable.^54^ The lignocellulose dopes prepared from samples *A*, *B*, and *C* of different lignin contents were dissolved in the IL (6% solid content). During the extrusion, the dopes strongly tend to gel (Fig. S3†); based on this observation, another sample of lower concentration was considered, sample *D* (Sample *D* = Sample *A* at half the concentration, 3%). Sample *D* allowed a wide liquid frequency window before the gelation point and lowered down the elastic and loss moduli of the dope (Fig. S3†). The dopes studied presented viscoelastic behavior at first. They were all liquid in the low-frequency region (loss modulus, *G*′′ > elastic modulus, *G*′). Moreover, depending on the composition, they reached the cross-over point (*G*′′ = *G*′ = G^*^) at a low frequency, behaving as gels afterward (*G*‘ > *G*′′). The cross-over point shows a linear dependency on the lignin composition and solid content (a higher lignin content correlated with a lower cross-over point frequency; similarly, the lower the solid content, the higher the cross-over point frequency).

LCF samples produced from the respective pretreated kraft samples (*A*, *B*, and *C* of 6% solid content) presented an average filament diameter after drying of 400(50) μm and underwent 60% shrinkage compared to the extrusion diameter for the three different samples. In contrast, sample *D* (3% solids) exhibited a 79% shrinkage. This extensive diameter reduction led to remarkable filament flexibility and soft surfaces (Fig. 3c–e). Note that minor fractures at the surface level can be attributed to the drying stress provoked by the presence of lignin, Fig. 3d.^55^ TheLCF*‘*s outstanding flexibility is attributed to the combined effect of well-aligned cellulose fibrils embedded in a reinforcing lignin matrix.^56,57^Fig. 3e–f show the typical SEM images of the LCF cross-section area where cellulose fibril bundles with preferential orientation in the flow direction were observed to be embedded in a lignin matrix. The fibril orientation was measured from Hermans’ orientation parameter using the WAXS azimuthal distributions of the (1–1 0) peak (Fig. 3g).

Interestingly, the lignin content did not affect the orientation of the fibrils. The average Herman's orientation parameter fluctuated between 0.21 and 0.26 for all the filaments measured (Table S4†). Other morphological parameters, such as X-ray diffraction peak positions, crystallinity index, and crystal size, did not track with lignin content (see Table S5† for peak positions, crystallinity index, and crystal width for all the filaments of raw pulp samples, respectively). We highlight that no significant differences between the samples (*A, B, C*, and *D*) were found in the regenerated and raw states (kraft pretreated), respectively. Fig. S4† includes the raw data, and Fig. S5† shows the peak's deconvolution analysis. The X-ray diffraction peaks for LCF exhibited the typical cellulose II type. In contrast, the raw pulp samples (after kraft pretreatment) and GVL-treated samples revealed the typical cellulose Iβ diffraction peaks.^58^ Additionally, due to the presence of lignin, a broad peak appeared at 2*θ* = 20° (wheat color),^59,60^ and the cellulose amorphous (green color) peak appeared at 2*θ* = 18° (corresponding to cellulose Iβ and 16° for cellulose II).^61^ The diffraction peaks for the GVL-pretreated samples (Fig. S5c†) revealed a decrease in crystallinity after regeneration, given the lower intensities of the (2 0 0) and (0 0 4) peaks.

**Fig. 4:**
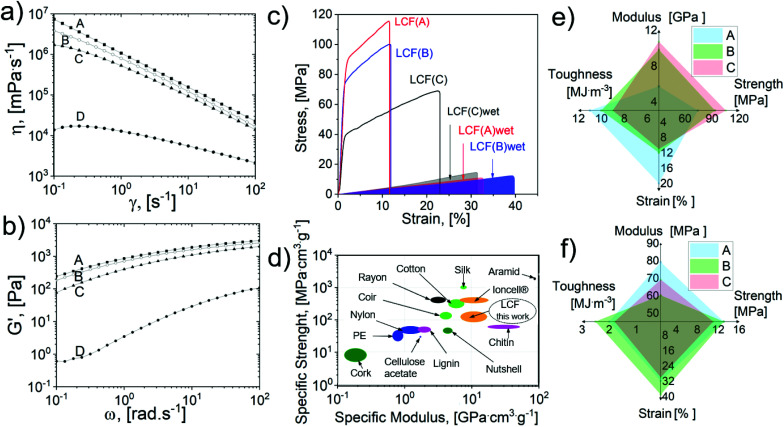
Dope rheology and mechanical performance of the regenerated LCF: (a) dynamic viscosities, (b) elastic modulus, (c) typical tensile test of LCF samples in dry and wet (shadow) states. (d) Ashby plot of the LCF and different biomaterials, plastics, and synthetic fibers.^54,66–70^ LCF's average (e) dry and (f) wet mechanical properties of samples *A*, *B* and *C*.

TGA analysis revealed the effect of the pulp kraft pretreatment: the samples with high lignin content yielded higher ash with sharper and wider DTG peaks attributed to lignin degradation products (Fig. S6†).^62^ These differences in lignin composition and fiber morphologies potentially impact the rheological and mechanical properties of the dopes and regenerated samples, and to study these effects, different rheological and mechanical properties were studied, Fig. 4.

Dynamic viscosities (*η*) and elastic modulus (*G*′) were assessed at different strains and frequencies, see Fig. 4a and b. As expected, sample *D* presented the lowest viscosity and storage modulus. The dopes with high lignin content presented a higher dynamic viscosity and storage modulus (sample *D versus C*). The dopes presented shear thinning behavior (drastic viscosity drop at an elevated shear rate).^63^ For instance, as shown in Fig. 4a, shear rates >10 s^−1^ promoted a drop of two orders of magnitude in the zero-shear rate viscosity for all the dopes. This shear rate value is well below the operational shear rate for the spinning and printing conditions (ESI, protocol 2.5†); consequently, under these optimal conditions, it is possible to promote a certain degree of fiber alignment to impact the regenerated filament's mechanical properties positively.^64^ As discussed previously, Herman's factor for the samples was not significantly different among the samples; however, the LCF samples’ mechanical performance in dry and wet states significantly depended on the lignin and solid content, Fig. 4c. Table 1 summarizes the results of mechanical tests of LCF samples; additional information on the LCF density, tenacity, and swelling diameter after water immersion (overnight) is also presented.

**Table 1:** LCF mechanical performance under dry and wet conditions

Sample	Modulus [GPa]	Strength [MPa]	Strain [%]	Toughness [MJ m^−3^]	Density [g cm^−3^]	Tenacity [cN dtex^−1^]	Diameter swelling [μm μm^−1^]
*A*	10.7(1)	105(8)	11(2)	9(2)	0.5(0.1)	2.3(0.3)	
*B*	9.8(1.3)	93(12)	12(3)	10(2)	0.6(0.1)	1.7(0.2)	
*C*	5.7(1.2)	74(14)	20(6)	11(4)	0.8(0.3)	1.3(0.4)	
*D*	5.5(1)	82(14)	8(3)	6(2)	1.0(0.1)	1.0(0.1)	
*A*-wet	69(10)^a^	11(1)	30(5)	1.8(0.3)			0.3(0.2)
*B*-wet	60(21)^a^	13(4)	39(6)	2.5(0.6)			0.2(0.1)
*C*-wet	79(23)^a^	13(2)	32(5)	2(0.7)			0.4(0.1)
*D*-wet	85(27)^a^	10(3)	15(2)	0.8(0.2)			0.4(0.2)

a Values multiplied by 10^3^ for easy comparison.

According to the results summarized in Table 1, the mechanical properties of the LCF did not significantly depend on fiber orientation and dynamic viscosities. A tensile strength of up to 105 MPa was reached for samples with the highest viscosity and storage modulus (sample *A*). In contrast, a lower lignin content promoted a higher tensile strain, up to 20% in the dry state (sample *C*) and 39% in the wet state (sample *B*). It is expected that a lower lignin content would produce tougher filaments; nevertheless, the tenacity analysis showed a significant correlation with the density; consequently, samples with high lignin content and low density exhibited tenacities up to 2.3 cN dtex^−1^, similar to the values reported for viscous textiles.^65^ Furthermore, an Ashby plot (see Fig. 4d) for the specific strength and modulus demonstrates a competitive mechanical performance compared to other man-made materials such as nylon, rayon, polyethylene (PE), and cellulose acetate^66–69^ (though higher tenacities have been reported for all cellulose-based filaments using ILs).^54,70^ In addition, the LCF exhibited other exceptional properties (see Fig. 4e and f), such as the tensile strain under wet conditions, reaching up to 39%, a higher value than those reported for filaments tested under similar conditions.^71^ Recently, antimicrobial and anti-odor textiles were reported by incorporating polyphenol coatings.^72^ Hence, the polyphenol-rich fractions produced in the first valorization route can be combined with regenerated filaments, representing a possible closed-loop production strategy.

Considering BPw as agricultural waste, the results indicate quite competitive filaments that simultaneously present a high lignin and phenolic content. In this context, further applications can be considered, especially by exploring other properties such as the mechanical performance in the wet state and light reflectance. Some applications are proposed in the following section.^73^

#### 3D printed materials

The optimal formulation for material production by spinning or direct-ink-writing depends on the targeted physical properties. For instance, the previous sections demonstrated that spun filaments from sample *A* met the standard tenacities of traditional textile yarns. However, different mechanical properties can be considered for other applications, depending on the specific need.^74^ This trade-off between mechanical properties and functionality is critical in producing materials for energy storage,^75,76^ smart textiles,^77^ biomedical patches,^74,78^ among others.

**Fig. 5:**
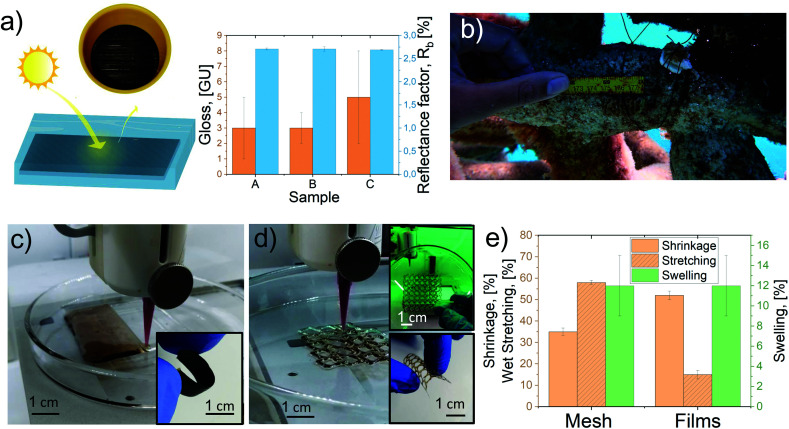
LCF and 3D printed samples’ properties and potential applications: (a) LCF surface, light reflectance factor, and gloss. (b) LCF used as a thread to fix underwater coral reef restoration supports. 3d-printed (c) flexible films and (d) customized patterned mesh. (e) Water stability of films and meshes.

Some uses for the LCF are shown in Fig. 5a and b together with 3D printed films, and mesh samples see Fig. 5c–e and supporting videos **Printing_film.mp4**, and **Printing_mesh.mp4**.†

Fig. 5a shows the construction of a material comprised of filaments placed parallelly and horizontally, developing a dark solid color, which was analyzed using the ISO-brightness, color, Tappi gloss 75 °C, and light reflectance factor (*R*_457_ or *R*_b_), Fig. S7† and Table S6.† The results suggested that it is possible to manufacture surfaces and textile fabrics with low gloss (less than 10%) and low reflectance (*R*_b_ < 3), independent of the lignin content. These properties (low gloss and radiance) are essential for textile camouflage,^79^ thermal protective clothing,^80^ and thermal energy textiles using phase change materials.^81^

The high stretchability of the filaments under wet conditions encouraged us to test LCF for underwater applications in marine environments. Here, we proposed LCF as a replacement for traditional plastics used to tie or hold parts for coral reef restoration.^82^ More specifically, LCF sample *A* was used in a Living Sea sculpture, a coral nursery project (Zoe),^83^ on the coast of Cozumel, Mexico. The materials were shown to be stable for several weeks and fully compatible with new coral implants before complete LCF biodegradation, Fig. 5b. The LCF supported new coral transplants on the mineral accretion structure for several weeks, a period long enough to allow integration by the growth of certain coral species to the structural ecosystem. The results associated with coral growth varied depending on the LCF thickness and the orientation and health of the coral specimen, which are subjects of ongoing research (Fig. S8†).

Finally, as proof of concept, the dope formulated with sample D was used to produce 2D printed films and customized 3D meshes, as shown in Fig. 5c, d and S9.†Fig. 5c shows the technique used to print 2 × 4 cm films (1.5 mm thickness) using parallel extruded adjacent lines that merged each other due to the low viscosity of formulation *D.* This technique allowed (after solvent exchange and drying) the production of robust and flexible films, as shown in Fig. 5c (inset image). Fig. 5d shows the production process of a 3D printed mesh 4 × 4 cm (0.5 mm thickness) with a customized pattern (Fig. S9†). Several small and large holes serve as the anchor point for potential applications such as water treatment^84^ and bioengineering devices.^85^Fig. 5d inset images present the solid meshes after drying, demonstrating the use of BPw lignocellulose residues for high fidelity and flexible structures, suitable in the design of materials with complex geometries.

The printed films and meshes presented comparable shrinkage to other reported materials^74,78^ (about 50% for films and 35% for meshes); moreover, the printing technology showed to be an attractive option for the production of robust and customized films and meshes with outstanding wet stretchability (up to 12% for films and 60% for patterned meshes, Fig. 5e).

## 4. Conclusions

Blueberry pruning waste (BPw) residues were used to produce added value platform chemicals such as sugars, antioxidants, and phenols. These chemicals are used by several industries, *e.g.*, pharmaceutical, food, biomedical, or serve as platforms for other biobased chemicals. These are not minor issues that deserve dedicated attention or analysis, which should also consider the screening of solvents,^86^ and refining techniques such as (supercritical) extraction^87^ and distillation^88^. From the lignocellulose fraction, filaments were produced by wet spinning and met the textile industry requirements. Hence, they were presented here as an option to “decarbonize” the operation. Finally, direct ink writing showed promising potential to transform BPw into advanced multidimensional structures for application in underwater environments and other potential uses. We demonstrate the technical opportunity and competitive prospects as far as material properties are concerned. Economic assessment requires further efforts to identify the uncertainties associated with data and scale and the integration of dynamic biomass market information, and techno-economic models.^89,90^

## Author contributions

G. Reyes contributed to the conceptualization, lead investigation, visualization, writing – original draft, and review & editing; C. M. Pacheco and A. González: data curation, visualization, and formal analysis; E. Isaza-Ferro and E. Pasquier: data collection and investigation; S. Alejandro-Martín and L. E. Arteaga-Peréz: investigation, validation, and review & editing; R. R Carrillo: investigation and methodology; I. Carrillo-Varela and R. T. Mendoça: methodology and review & editing; C. Flanigaan: investigation; O. Rojas: funding acquisition, resources, validation, and writing – reviewing & editing.

## Conflicts of interest

There are no conflicts of interest to declare.

## Supplementary Material

GC-024-D2GC00573E-s001

GC-024-D2GC00573E-s002

GC-024-D2GC00573E-s003

GC-024-D2GC00573E-s004
