# Supplementary material for: Upcycling agro-industrial blueberry waste into platform chemicals and structured materials for application in marine environments
Source: Green Chem. 2022 Apr 7;24(9):3794–804. doi: 10.1039/d2gc00573e (PMC9086861; doi:10.1039/d2gc00573e)
Supplement: GC-024-D2GC00573E-s004 [file GC-024-D2GC00573E-s004.pdf]

Electronic Supplementary Material (ESI) for Green Chemistry.
This journal is © The Royal Society of Chemistry 2022

SUPPORTING INFORMATION

Upcycling agro-industrial blueberry waste into platform chemicals and structured materials for application in marine environments

Guillermo Reyes,[*,a] Claudia M. Pacheco,[b] Estefania Isaza-Ferro,[c] Amaidy González,[d] Eva Pasquier,[a,e] Serguei Alejandro-Martín,[f] Luis E. Arteaga-Peréz,[d] Romina R. Carrillo,[g] Isabel

[a.] *Biobased Colloids and Materials, Department of Bioproducts and Biosystems, School of Chemical Engineering, Aalto University, FI-00076, Espoo, Finland*
[b.] *Facultad de Ingenierías, Universidad Cooperativa de Colombia, Cra 22 No. 7 – 06 sur, Villavicencio, Colombia*
[c.] *Department of Bioproducts and Biosystems, School of Chemical Engineering, Aalto University, FI-00076, Espoo, Finland*
[d.] *Laboratory of Thermal and Catalytic Processes, Facultad de Ingeniería, Universidad del Bío-Bío, Av. Collao 1202, Concepción, Chile*
[e.] *Université Grenoble Alpes, CNRS, Grenoble INP (Institute of Engineering), LGP2, F-38000 Grenoble, France*
[f.] *Laboratorio de Cromatografía Gaseosa y Pirólisis Analítica, Departamento de Ingeniería en Maderas, Universidad del Bío-Bío, Av. Collao 1202, Casilla 5-C, Concepción, Chile*
[g.] *Facultad de Ciencias Químicas, Depto. Química Analítica e Inorgánica, Universidad de Concepción, Concepción, Chile.*
[h.] *Laboratorio de Recursos Renovables, Centro de Biotecnología, Universidad de Concepción, Concepción, Casilla 160-C, Concepción, Chile*
[i.] *Centro de Investigación de Polímeros Avanzados, CIPA, Avenida Collao 1202, Edificio de Laboratorios, Concepción 4030000, Chile.*
[j.] *Facultad de Ciencias Forestales, Universidad de Concepción, Casilla 160-C, Concepción, Chile*
[k.] *Zoe – A Living Sea Sculpture in Cozumel, Av. Rafael E. Melgar, 77688 San Miguel de Cozumel, Q.R., Mexico*
[l.] *Bioproducts Institute, Department of Chemical & Biological Engineering, Department of Chemistry and Department of Wood Science, 2360 East Mall, The University of British Columbia, Vancouver, BC V6T 1Z3, Canada.*

Carrillo-Varela,[h] Regis Teixeira Mendonça,[i,j] Colleen Flanigan,[k] Orlando J. Rojas,[*,a,l]

1. FIGURES AND TABLES

2. PROTOCOLS

2.1 Liquid fraction analysis by High-Performance Liquid Chromatography (HPLC)

2.2 Derivatized liquid fraction analysis by Gas Chromatography-Mass Spectrometry

2.3 Analytical Pyrolysis coupled to Gas Chromatography-Mass Spectrometry (Py-GC/MS) and FTIR-ATR

2.4 Kraft pretreatment

2.5 Wet spinning and 3D-printing

3 ANALYTICAL METHODS

3.1 Rheology

3.2 Wet Spinning and 3D printing

3.3 Tensile test, morphology (SEM), Wide Angle X-ray Scattering (WAXS), and thermogravimetric Analysis (TGA-DTG)

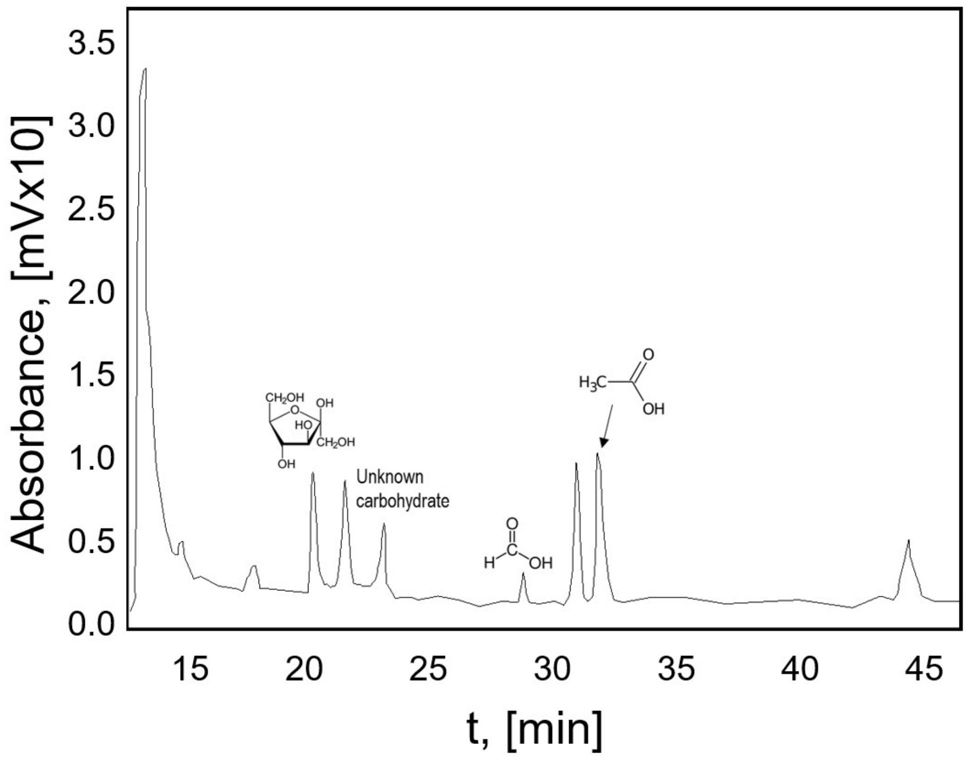

Figure S1. HPLC-UV/RID chromatogram for the GVL-extracted sample

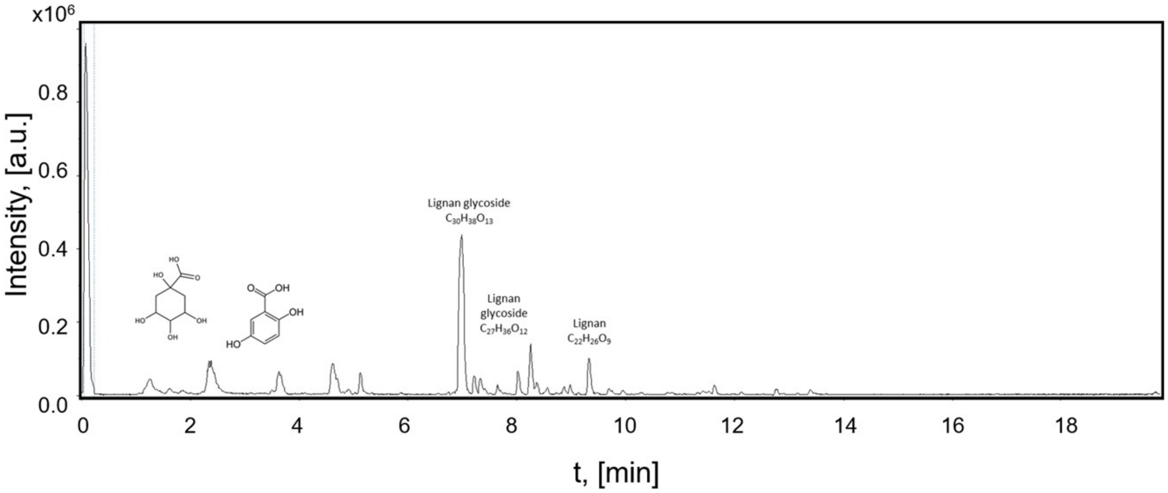

Figure S2. UHPLC-MS/MS chromatogram for the GVL-extracted sample

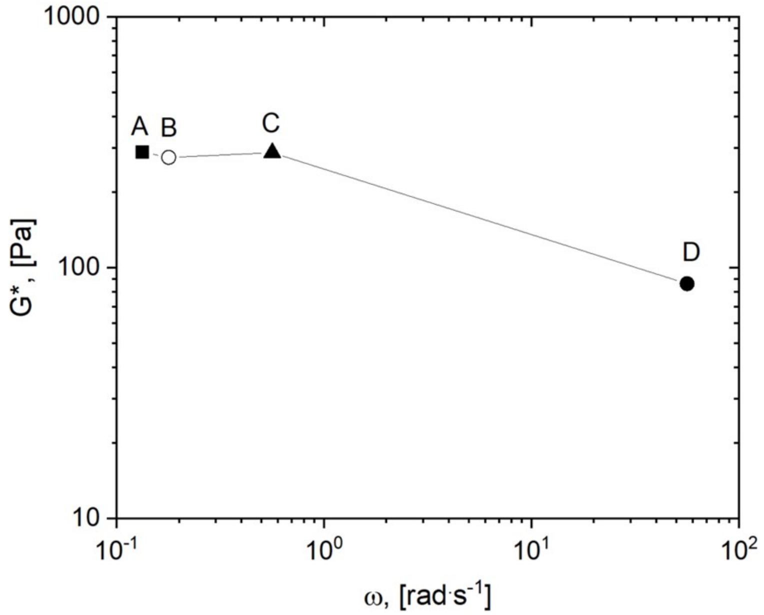

Figure S3. Cross over point (G' = G") in dopes rheology frequency swept

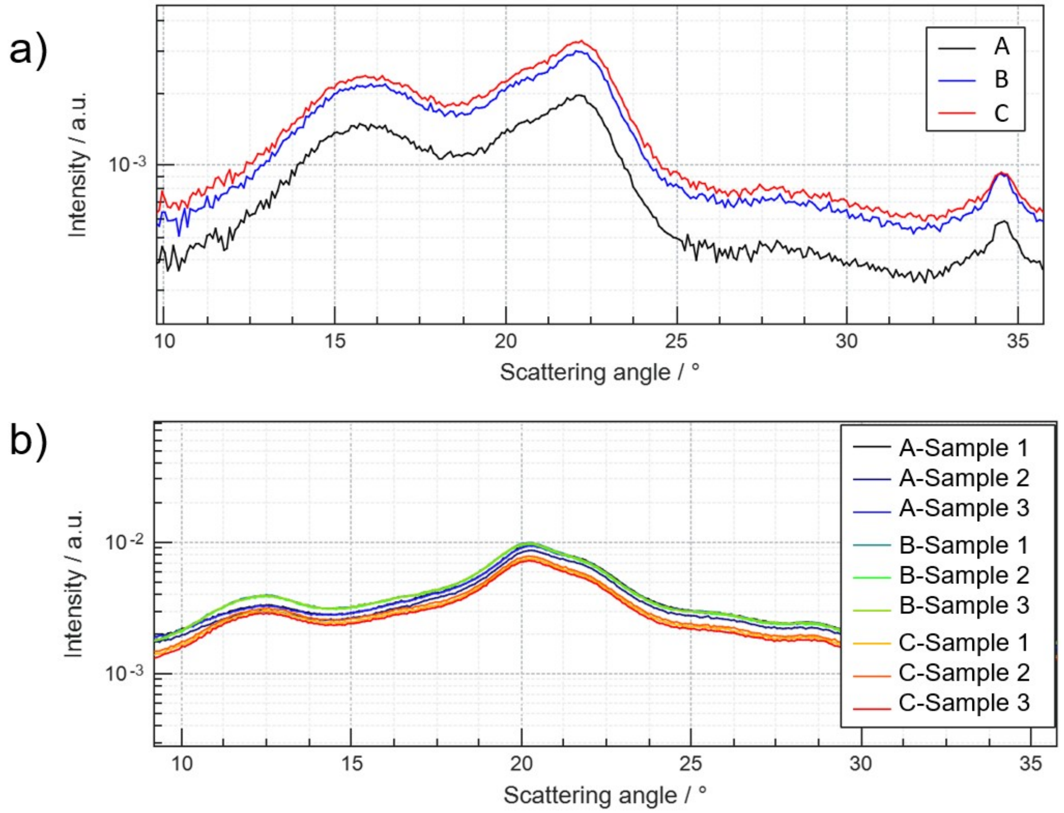

Figure S4. Raw data on X-ray diffraction patterns for pretreated and LCF samples: a) kraft pretreated pulp samples. b) filament samples LCF measured at three different positions.

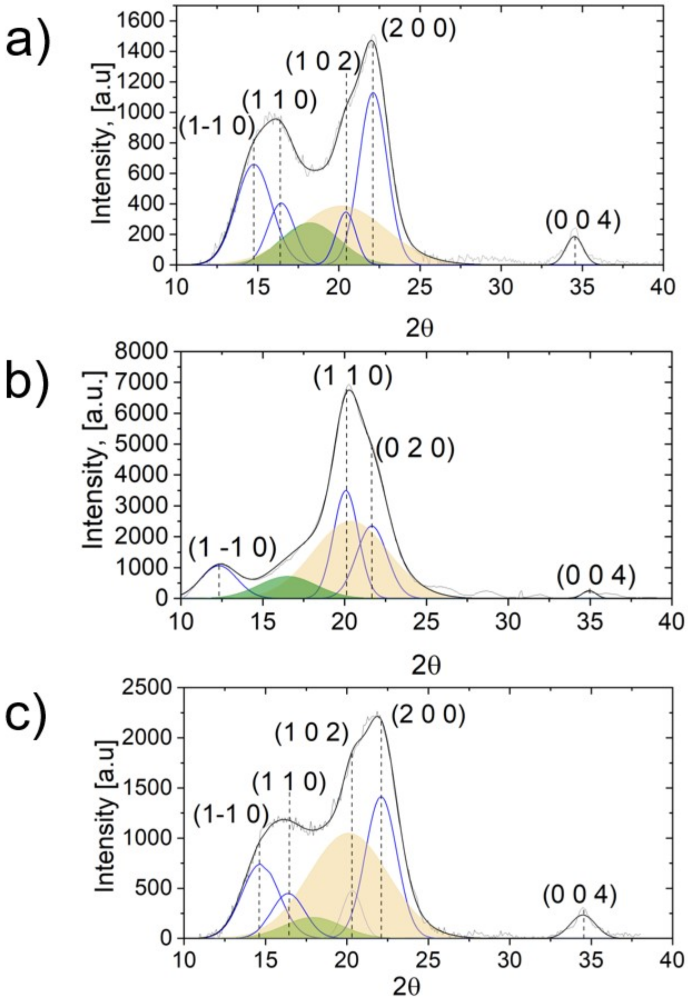

Figure S5. Pretreated and regenerated pulp samples diffraction peaks deconvolution analysis: a) pretreated kraft samples. b) regenerated LCF. c) GVl pretreated samples. The lignin peaks are shown with wheat shadow color and amorphous cellulose contribution with green shadow color.

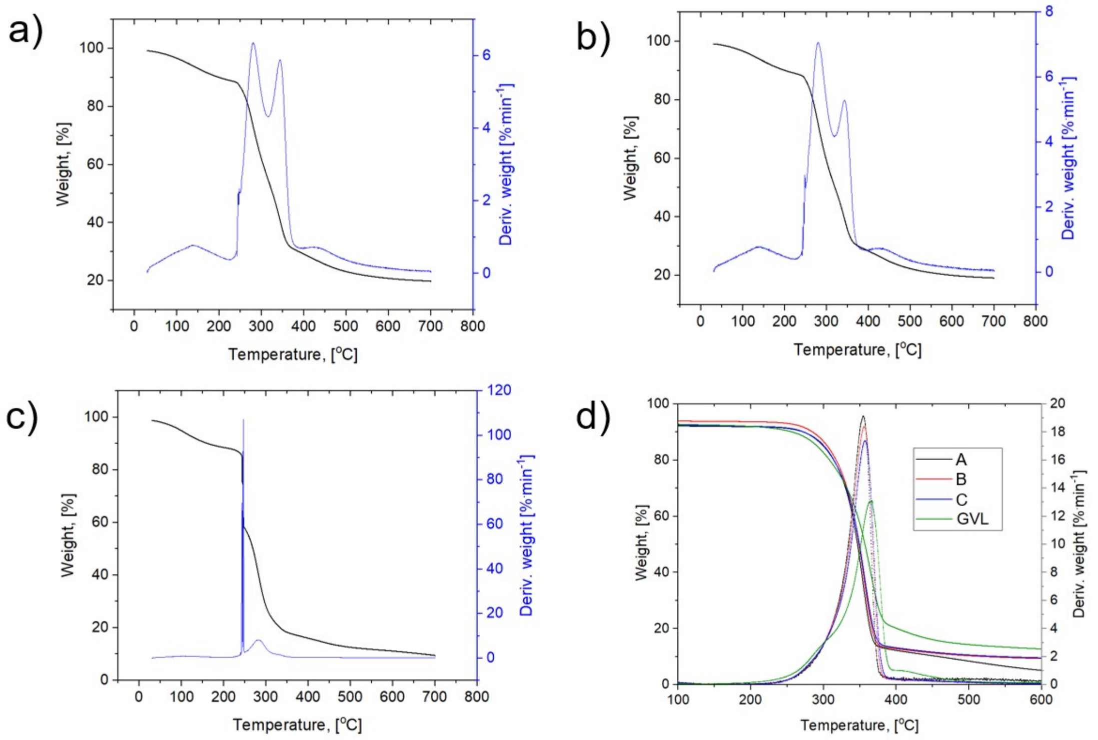

Figure S6. TGA and DTG analysis of pretreated pulps and LCF samples: a) Filament sample *A*. b) Filament sample *B*. c) Filament sample *C*. d) pretreated kraft sample pulps

The weight loss for all samples indicates degradation in the temperature range tested, exhibiting a weight loss > 80 % for temperatures above 200 °C. *LCF* samples showed two degradation steps; attributed to cellulose and lignin degradation products, respectively.[1] The main peak attributed to cellulose degradation appears above 200 °C with a maximum of 280 °C; this peak merges with the lignin degradation peak at around 350 °C. From Figure S6 is evident that the lignin peak becomes sharper and wider for samples with higher lignin content; moreover, the ash content for LCF samples (A, B, C) were 20, 19, and 9 %, respectively. In contrast, the pretreated pulp samples (Figure S6d) exhibited the same trend as the regenerated samples but with a peak that overshadowed the cellulose degradation peak attributed to hemicelluloses and lignin. The lignin peak appears shifted around 10 °C compared to the LCF samples (maximum around 360 °C).

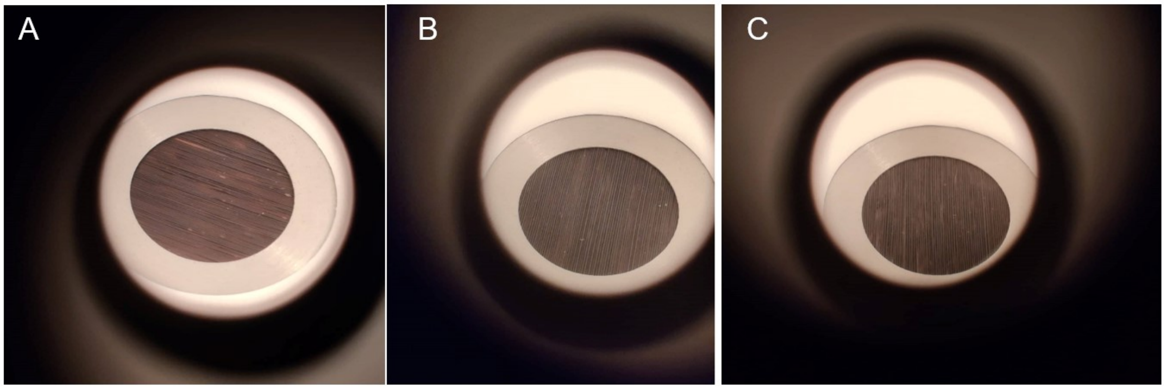

Figure S7. *LCF* sample surfaces A, B, and C used for color, gloss, and iso-brightness tests

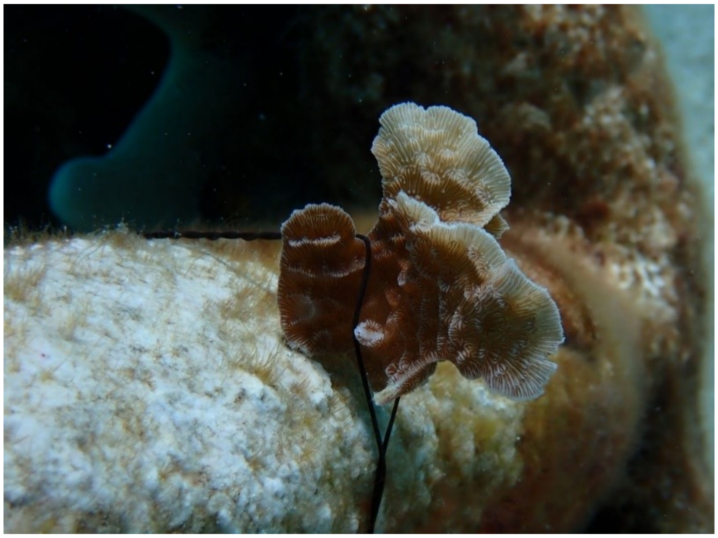

Figure S8. Coral sample fixed by twisting *LCF*

LCF for Coral reefs restoration.

LCF from sample *A* were used to tie fragments of the coral species *Agaricia tenuifolia* to the electrolytic mineral accretion coral habitat aka artificial reef.[2] The strength and pliability of the filament for one month were monitored to verify that these filaments will support multiple applications for coral and marine biodiversity regeneration. The purpose is that the coral will cement itself to the structure, and in time when the filament biodegrades, it will not add pollutants to the ocean environment.

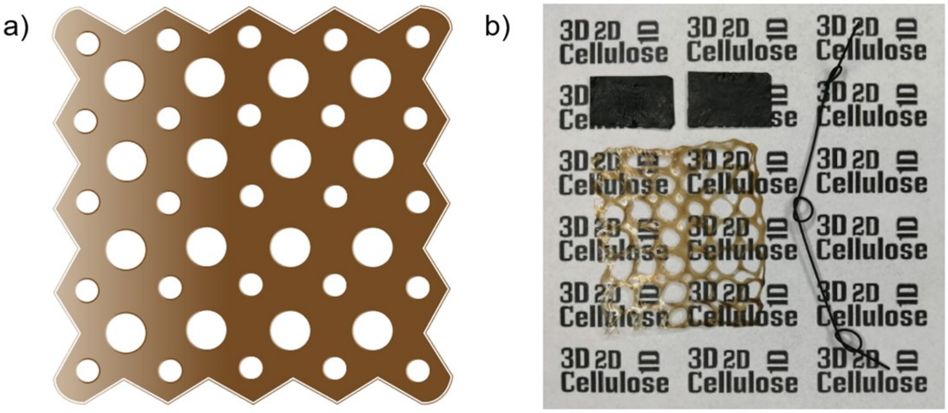

Figure S9. 3D printed mesh patterned samples, a) 3D printed pattern, b) dry films, mesh, and filament samples

Table S1. Chemical compounds detected by Py-GC/MS of BPw and fractionated samples

Family	Compound	BPw	CRSF	LRSF
Phenols	Phenol, 2,6-dimethoxy-	3,2	1,8	7,7
	2-Methoxy-4-vinylphenol	2,0	2,1	5,5
	Phenol, 4-ethenyl-2,6-dimethoxy-	0,7	1,1	2,3
	Creosol	2,8	1,7	2,5
	(E)-2,6-Dimethoxy-4-(prop-1-en-1-yl)phenol		0,8	1,2
	Phenol, 2-methoxy-	1,9	0,9	4,8
	Phenol, 2-methoxy-4-(1-propenyl)-	1,6	1,1	1,3
	Phenol, 4-ethyl-2-methoxy-	0,6		1,9
	5-tert-Butylpyrogallol			2,1
	Phenol, 2,6-dimethoxy-4-(2-propenyl)-		0,3	0,5
	Eugenol	0,3	0,3	
	Subtotal	***13***	***10***	***30***
Acids	Acetic acid	18,0	15,5	12,4
	Propanoic acid, 2-oxo-, methyl ester	3,4	2,9	
	Acetic acid, (acetyloxy)-	6,1	5,2	
	Acetic acid ethenyl ester	2,1	1,3	
	Carbonocyanidic acid, ethyl ester	0,8	1,0	
	Butanedioic acid, cyclic hydrazide		0,8	
	Propanoic acid, 2-oxo-			1,0
	Subtotal	***30***	***27***	***13***
Aldehydes	Methyl glyoxal	4,6	4,4	2,1
	Furfural	3,0	3,9	1,6
	Acetaldehyde, hydroxy-	2,2	1,8	0,0
	Succindialdehyde	2,5	1,0	0,0
	Acetaldehyde, (3,3-dimethylcyclohexylidene)-, (Z)-		0,7	0,0

	2-Furancarboxaldehyde, 5-methyl-		0,7	0,0
	Subtotal	***12***	***13***	***4***
Ketones	2-Propanone, 1-hydroxy-	6,3	3,2	
	2-Cyclopenten-1-one, 2-hydroxy-	4,0	2,2	
	1,2-Cyclopentanedione, 3-methyl-	2,1	2,2	
	2(5H)-Furanone	2,0	0,8	
	2-Propanone, 1-(acetyloxy)-	1,2	0,7	0,6
	3-Pentanone	1,3	0,6	
	2-Cyclopenten-1-one, 2-hydroxy-3-methyl-		1,6	
	Ethanone, 1-oxiranyl-		1,4	
	2-Butanone	0,9		0,5
	Acetone			1,2
	6,6,9a-Trimethyl-decahydronaphtho[1,2-c]furan-1,4-dione			0,9
	2(5H)-Furanone, 5-methyl-		0,5	
	2(3H)-Furanone, 5-heptyldihydro-			0,4
	4-Methyl-5H-furan-2-one		0,3	
	2,3-Butanedione			0,6
	Subtotal	***18***	***14***	***4***
Alcohols	1-Decanol		0,4	1,3
	1,2-Ethanediol, monoacetate		6,1	1,0
	2-Furanmethanol	1,2	1,4	
	2-Pentanol, 5-(2-propynyloxy)-		0,9	
	Furaneol		0,4	
	1-Undecanol		0,3	
	Subtotal	***1***	***10***	***2***
Amines	Oxazolidine, 2,2-diethyl-3-methyl-	3,9	9,0	
	Formamidoxime		1,0	
	Benzonitrile, m-phenethyl-			1,4
	Subtotal	***4***	***10***	***1***
Sugars	1,6-Anhydro-α-d-galactofuranose		0,6	
	1,3-Di-O-acetyl-α-β-d-ribopyranose		0,7	
	L-Glucose		0,6	
	Subtotal		***2***	
HC_Aromatics	3,5-Dimethoxy-4-hydroxytoluene	1,3	1,7	5,5
	Subtotal	***1***	***4***	***6***
Ethers	Furan, tetrahydro-2,5-dimethyl-, trans-(±)-			8,7
	Butane, 1,2:3,4-diepoxy-, (±)-	0,9	0,6	
	Benzene, 1,2,3-trimethoxy-5-methyl-			2,2
	Furan, tetrahydro-2,5-dimethyl-			1,2
	Di-n-propyl ether			0,6
	Subtotal	***1***	***1***	***13***
Unknown	unknown	6,9	0,3	23,2
	**Total**	**100**	**100**	**100**

Table S2. Main compounds detected by der-GC/MS (reference to Figure 2c)

Peak	Retention time (min)	Compound	A (%)
1	27.185	Cyclohexasiloxane, dodecamethyl-	3.53
2	33.483	2-Methoxy-4-vinylphenol	1.35
3	35.375	5-Hydroxymethylfurfural	3.62
4	47.826	(3,5-dimethoxyphenyl)-hydroxyacetic acid	2.82
5	56.687	l-(+)-Ascorbic acid 2,6-dihexadecanoate	7.28
6	62.023	cis-Vaccenic acid	20.51
7	69.503	9-Octadecenamide, (Z)-	9.71
8	71.527	Tetratriacontane	8.81
9	79.151	13-Docosenamide, (Z)-	7.18
10	90.927	D-Friedoolean-14-en-3-one (Taraxerone)	14.86
11	91.833	beta.-Amyrin	3.79

Table S3. Effect of active alkali on the yield, kappa number, physical and optical properties of BPw pulps.

Sample	Alkali (%)	Pulp Yield (%)	Kappa number	Viscosity ($ml \cdot g^{-1}$)	Fiber Length (mm)	Fiber width (µm)	Brightness ISO (%)
A	22	35.0	31	1081	0.509	20.8	24.1
B	24	33.5	23	1070	0.504	19.7	27.0
C	26	34.8	17	980	0.503	19.6	28.6

Table S4. Anisotropy angle and Herman's factor measured for *LCF* samples with three repetitions each

Sample	No.	Anisotropy angle	Herman's factor
A	1	168	0.17
	2	170	0.30
	3	155	0.30
		Average	0.26
		Standard deviation	0.07
B	1	173	0.29
	2	165	0.23
	3	164	0.13
		Average	0.22
		Standard deviation	0.08
C	1	173	0.28
	2	160	0.13
	3	175	0.22
		Average	0.21
		Standard deviation	0.07
D	1	170	0.29
	2	163	0.22
	3	164	0.14
		Average	0.22
		Standard deviation	0.08

Table S5. Filaments and raw pulps WAXS analysis

Sample	Type	Peak position (deg)					τ[Å]	CI [%]
		(1-10)	(110)	(102)	(200)	(020)		
Regenerated Filaments (*A, B, C, D*)	II	12.3	20.2			21.7	41(6)	69(12)
Raw pulp (*A, B, C*)	Iβ	14.7	16.5	20.4	22.1		44(11)	75(20)
GVL treated	Iβ	14.7	16.4	20.3	22.1		39(12)	85(25)

Table S5 presents the *LCF* cellulose sample's diffraction peaks positions, crystallinity index (CI), and crystallite lateral width (τ).

Table S6. Tappi 75 ^{o} gloss measurements on LCF surfaces and printed films

Sample No.	Position [deg]	Surface filaments A	B	C	Printed film D
1	0	5,4	5,7	10	7,7
2	0	6	5,8	10	7,8
3	0	6,3	6,7	10,7	8,3
4	0	5,1	5,9	7,7	10
5	0	6,4	5,8	12,7	12
6	45	2,4	2,9	4,3	7,6
7	45	2,8	2,6	3,8	7,7
8	45	2,3	2,8	4	8,3
9	45	2,4	3,1	3,3	7,9
10	45	2,2	2,7	3,3	8,5
11	90	1,7	2,3	2,7	10,6
12	90	1,8	2,1	2,7	9,7
13	90	1,7	2,1	2,6	8,2
14	90	1,7	1,7	2,5	7,2
15	90	1,7	1,8	2,8	7,7
16	135	2,6	2,8	3,3	
17	135	2,4	2,8	3,2	

18	135	2	2,7	3,4
19	135	2,2	2,8	3,7
20	135	2,2	2,8	3,6

2. PROTOCOLS

2.1 Liquid fraction analysis by High-Performance Liquid Chromatography (HPLC)

The HPLC-UV-RID analyses were carried out in a Shimadzu HPLC system equipped with SPD-20AV UV-Vis detector and RID-10A refractometer (Shimadzu Corporation, Kyoto, Japan). Data processing was performed using Lab-Solutions software (version 1.25 Shimadzu, Japan). The chromatographic method was performed using two Rezex ROA-Organic Acid H^+ (8 %), 300 x 7.80 mm (Phenomenex, Torrance, C.A. USA). The mobile phase in isocratic mode consisted of H_2SO_4 0.0075 mol·L^{-1} in deionized water with a flow rate 0.6 ml·min^{-1}. The UV-Vis detector (210 nm) and the refractometer were serially connected. Also, the samples were analyzed by LC-MS/MS in Nexera X2 UHPLC system equipped with SPD-M20A diode array detector (DAD) and LCMS-8030 triple quadrupole (TQ) mass spectrometer (Shimadzu Kyoto, Japan). The system was controlled by Shimadzu LabSolutions software (version 5.8, Shimadzu, Japan). Separation was carried out on Phenomenex (Torrance, CA, USA) Kinetex XB Core-Shell C18 column (100 mm x 4.6 mm, 2.6 µm), thermostated at 30 °C, using a mobile phase composed of 50% ultrapure water with 0.5 % (v/v) formic acid and 50 % acetonitrile, at a flow rate of 0.7 mL·min^{-1}. Full scan spectra were acquired from m/z 50 to 1000.

2.2 Derivatized liquid fraction analysis by Gas Chromatography-Mass Spectrometry (der-GC/MS)

The GC analyses were performed with a gas chromatograph (GC-2010 Plus, Shimadzu) equipped with a single quadrupole mass spectrometry detector (QP 2010 Ultra, Shimadzu). The compounds were separated in a HP-5ms capillary column (60 m x 0.25 mm x 0.25 μm, Agilent Technologies, USA). The injector and detector temperatures were kept at 250 °C and 280 °C, respectively. The initial GC oven temperature was 45 °C which was held for 4 min before heating to 280 °C at 3 °C min^{-1}. The final temperature was maintained for 40 min.

Before GC injection, the samples were derivatized with BSTFA + 1% TMCS reagent (Sigma Aldrich, Germany) by 3 h at 70 °C with a controlled and constant flow of N_2 (sample concentrator SC II, Biobase, China).[3]

2.3 Analytical Pyrolysis coupled to Gas Chromatography-Mass Spectrometry (Py-GC/MS)

The solid samples from chemical fractionation (lignin-rich and cellulose-rich fractions in Fig. 1) will be used as feedstock for pyrolysis in a micropyrolysis unit (CDS Analytical Pyroprobe (5200 HPR), coupled to Gas Chromatograph (Clarus 690, Perkin-Elmer) equipped with a mass spectrometer (Clarus SQ8T). In a typical experiment, a half milligram of solid samples (CRSF or LRSF) were pyrolyzed for 12 s at 500 °C using He as a carrier gas. Identification of compounds was made by comparing the chromatographic results (Turbo Mass, v6.1.2.2048) with the National Institute of Standards and Technology database (NIST 2017 v2.3). The abundance of each species was calculated by applying a linear proportionality between the area of the chromatographic peaks corresponding to a particular compound and its relative concentration. Based on this principle, the selectivity was

estimated by the ratio between the chromatographic peak area of a *ith*-compound ($PArea_i$) and the sum of total detected peak areas ($\Sigma PArea_i$) (**Equation S1**).

$$S_i = 100 \times \left(PArea_i \Big/ \sum_{i=1}^{n} PArea_i \right) \tag{S1}$$

Solid fractions from solvolysis were characterized by Fourier-Transform Infrared Spectroscopy (**FTIR**). The IR spectra were recorded over 32 scans in the range 4000 cm^{-1} to 400 cm^{-1}, with a resolution of 2 cm^{-1} using a Nicolet is20 spectrometer (Thermofisher, USA) and a Specac Quest ATR accessory equipped with a Ge crystal (Specac, UK). The spectra were post-processed and analyzed using the Omni Specta software (v2013).

2.4 Kraft pretreatment

The kraft process allows the fragmentation and solubilization of lignin to obtain cellulosic pulp using a solution of sodium hydroxide (NaOH) and sodium sulfide (Na_2S), expressed as active alkali and sulfidity in NaOH basis. BPw were cut into chips (small pieces of 3 cm to 5 cm in length). The cooking of BPw was performed in a Parr reactor 4848 (volume 1l) that was loaded with 100 g of chips (dry basis) and 600 ml of white liquor with variable active alkali (22 %, 24 %, and 26 %), sulfidity (both expressed in NaOH basis), corresponding to samples *A, B, C* respectively. The cooking temperature was 165 °C and H-factor of 800. After each reaction, the black liquor was drained, the pulp was washed with abundant tap water, screened for shives removal in a 0.2 mm slot screen followed by the determination of the screened pulp yield.[4] The Kappa number (KN) that expressed residual lignin in pulps was determined according to TAPPI T236 om-99 standard. The fiber dimensions (fiber length and fiber width) were obtained by analysis of two-dimensional images in a FiberTest equipment (Lorentzen & Wettre, Sweden) following the methodology reported by Carrillo *et*

al.[5] The viscosity of pulps (related with the degree of polymerization (DP) of cellulose) was determined by cupriethylenediamine (CED) dissolution in a capillary viscometer according to TAPPI T230 0m-04 TAPPI 222. The ISO brightness of pulps was determined according to the ISO 2470 standard using a spectrophotometer Elrepho (Lorentzen & Wettre SE 070).

3. ANALYTICAL METHODS

3.1 Rheology properties

The shear rheology of the dissolved lignocellulose material was monitored in the steady and oscillatory modes using an Anton Paar Physica MCR 302 (Anton Paar GmbH, Austria) rheometer equipped with a Peltier hood H-PTD 200 for controlled temperature and humidity; all the measurements were performed at 60 ^{o}C. The tests were carried out with a parallel plate geometry of 25 mm diameter and 1mm gap, viscosity measurement, frequency swept study at 0.1 % amplitude, and cross-over point was studied.

3.2 Wet spinning and 3D-printing

Kraft pulp pretreated samples and IL [emim][OAc](CAS No. 143314-17-4, purity = 97 %, Sigma Aldrich) were vacuum dried overnight (60 ^{o}C, 200 mbar) and used to prepare dissolved dopes at 6 % w/w. The dopes at 6 % w/w were produced in a stainless-steel reactor under vacuum (50 mbar, 120 ^{o}C, 3 h, 30 rpm) for samples (*A*, *B*, *C*). Sample *D* was obtained from sample *A* at 3 % w/w using the same dissolving conditions. The lignocellulose filaments or *LCF* were obtained by the wet spinning of the corresponding samples dopes. The dopes solid content concentrations were selected for practical reasons since concentrations above 6 % prevented a proper mixing during the dissolution process due to the high viscosity. The sample *D* at 3 % w/w was prepared specially for the 3D printing process since lower

viscosities were required for a proper extrusion on the device. The dopes were homogenous, and a filtration step (50 µm mesh) was included before the spinning and 3D printing steps.

The dopes were extruded using stainless steel syringe (CHEMYX, SS050, USA) equipped with a spinning nozzle (Ramé-Hart Instrument CO, gauge 17, inner diameter Φ_i = 1.07 mm), the syringe temperature was maintained at (120 ^oC) with a heating jacket provided from the same company. The pump (CHEMYX, Model FUSION 6000, USA) was used to extrude the dope into a water bath for the LCF regeneration (Milli Q® type I water from Merck (2 liters, 25 ^oC)). The system was operated at 1 $mL \cdot min^{-1}$, and the filaments were collected in 6 cm diameter silicon roller without stretching (constant draw ratio of D_w = 1.0). The collected filaments were cut into small pieces (0.5 m) and dried for 24 h at room conditions in a metallic board holding the filament's ends with magnets.

The 3D printing process was carried out in BIO X Bioprinter (CELLINK, Gothenburg, Sweden) equipped with a pneumatic print head. All printed samples had a rectilinear infill pattern and 20 % infill density equipped with a clear pneumatic 3 mL syringe with nozzle 20 G (630 µm). 2D films 20 x 40 x 1.5 (mm) were printed (print head parameters: 60 ^oC, 65 kPa, 5 mm/s) and 3D patterned meshes (print head parameters: 60 ^oC, 50 kPa, 4.5 mm/s) over glass petri dishes. The printed samples were subjected to solvent exchange with milli Q® water for 1 hour, and later the regenerated material was dried between two fabrics (SEFAR NITEX® fabric, code: 03-1/1) in a cold press (25 ^oC, 1 kPa).

The shear rates during the wet spinning and 3D printing process can be calculated using plug flow approximation.[6] For the spinning conditions, the shear rate is approximately 35 $s^{-1,}$ and for the printing process, 8 s^{-1}.

The filaments and 3D printing structures drying and stretching properties were calculated according to the following equations:

$$shrinkage = \frac{L_{wet} - L_{dry}}{L_{wet}} x100 \tag{S2}$$

$$swelling = \frac{L_{wet} - L_{dry}}{L_{dry}} x100 \tag{S3}$$

$$stretching = \frac{L_{extended} - L_{not\ extended}}{L_{not\ extended}} x100 \tag{S4}$$

where *L* is the material reference dimension (for *LCF* diameter), **Equation S2** was used to measure the LCF, and 3D printed materials shrinkage after drying with reference to the freshly extruded material dimensions, **Equation S3** was used for measuring the swelling after overnight immersion in water of the previously dried materials. The stretching percentage was measured after extending the material and analyzing its final length before the break (using the tensile test equipment).

3.3 Mechanical performance, structure, and thermal behavior

LCF's mechanical properties were studied using a Universal Tensile Tester Instron 4204, 100 N load cell, test speed 5 mm/min. According to the ASTM D3822/D3822M standard, samples were prepared and analyzed. The thickness of dry and wet samples (immersed in deionized water overnight) was measured using a digital micrometer (Mitutoyo, Japan) and repeated five times in different positions; wet samples were measured by placing the samples between glass slides.[7] Ten replicas of each sample were taken for the mechanical tests. Surface morphology was analyzed using SEM (JEOL JSM-7500FA, Germany) at the Nanomicroscopy Center in Aalto University, Finland. The SEM possesses a 0.6 - 1.4 nm resolution at 30 - 1 kV. Before imaging, the samples were vacuum dried and fractured using liquid nitrogen (measuring cross-section areas). The samples were sputtered using a sputtering device (Emitech K350, Quorum Technologies Ltd, UK) operating at 220 v -50 Hz

– 10 A for 1.5 min with an Au/Pt coater disc, obtaining layers of ~ 10 nm Au/Pt over the samples. The images were analyzed using SEM software and ImageJ.[8]

Sample's orientation, crystallinity index, and crystallite lateral size (2 0 0) were obtained, using a bench beamline equipment SAXS/WASX device (Xenoxs, Xeuss® 3.0, UK) in the transmittance mode. Angular scanning was conducted from 5 to 50 ° at 5 °/min with Cu Kα radiation (1.54 Å). The generator was working at 45 kV and 200 mA. Background correction due to the sampler holder and the air was made by subtracting the sample diffractogram data with the corresponding blank data (without sample). Every sample was measured in three different positions, the LCF was held with scotch tape, and the powder samples were measured in pressed discs.

The deconvolution procedure (Gaussian functions) was performed using fityk.[9] Herman's orientation parameter was calculated from the integration of the azimuthal intensities according to **Equation S5**.[10]

$$f = \frac{3}{2}\int_{0}^{\pi} cos^2\varphi \cdot \tau(\varphi) \cdot sin\varphi d\varphi - \frac{1}{2} \tag{S5}$$

Where φ is the azimuthal angle and τ(φ) represents the normalized azimuthal intensities distribution after subtraction the isotropic contribution. The crystallite lateral size τ(Å) was calculated by the Scherrer equation.[11]

$$\tau = \frac{K\lambda}{\beta \cos\theta} \tag{S6}$$

Where K is the Scherrer constant (0.94), λ is the wavelength of the X-ray radiation (1.54059 Å), β is the FWHM (Full width at half maximum) of the diffraction peak in radians, and θ is the diffraction angle of the peak. The Segal crystallinity index (CI) was calculated according to the following equation[12]:

$$CI = \frac{I_t - I_a}{I_t} * 100 \tag{S7}$$

where I_t is the total intensity of the (0 0 2) peak for cellulose *I* and I_a is the intensity assigned to the amorphous cellulose.

The thermal stability of the samples was studied using a thermogravimetric analyzer (sensitivity of 0.001 mg) (TA instruments, Q500, USA). Around 10 mg of each sample were placed in the thermogravimetric analysis under Nitrogen flow (60 ml/min). The programmed temperature procedure is maintained at 30 °C for 15 min, then increased to 700 °C (ramp rate 10 °C·min^{-1}) and finally kept at 700 °C for 30 min.

REFERENCES

1 T. Sebio-Puñal, S. Naya, J. López-Beceiro, J. Tarrío-Saavedra and R. Artiaga, *J. Therm. Anal. Calorim.*, 2012, **109**, 1163–1167.

2 C. Flanigan, Zoe a living sea sculpture, https://zoecoral.com/.

3 A. I. Ruiz-Matute, O. Hernández-Hernández, S. Rodríguez-Sánchez, M. L. Sanz and I. Martínez-Castro, *J. Chromatogr. B Anal. Technol. Biomed. Life Sci.*, 2011, **879**, 1226–1240.

4 P. Reyes, A. Ferraz, M. Pereira, J. Rodríguez and R. T. Mendonça, *Holzforschung*, 2015, **69**, 33–40.

5 I. Carrillo, S. Valenzuela and J. P. Elissetche, *IAWA J.*, 2017, **38**, 105–116.

6 M. J. Lundahl, V. Klar, L. Wang, M. Ago and O. J. Rojas, *Ind. Eng. Chem. Res.*, 2017, **56**, 8–19.

7 B. D. Mattos, B. L. Tardy, L. G. Greca, T. Kämäräinen, W. Xiang, O. Cusola, W. L. E. Magalhães and O. J. Rojas, *Sci. Adv.*, 2020, **6**, 1–11.

8 National Institutes of Health, ImageJ, https://imagej.nih.gov/ij/index.html, (accessed 22 May 2017).

9 M. Wojdyr, *J. Appl. Crystallogr.*, 2010, **43**, 1126–1128.

10 J. J. Hermans, P. H. Hermans, D. Vermaas and A. Weidinger, *Recl. des Trav. Chim. des Pays-Bas*, 1946, **65**, 427–447.

11 P. Scherrer, *Nachrichten von der Gesellschaft der Wissenschaften zu Göttingen, Math. Klasse*, **1918**, 98–100.

12 L. Segal, J. J. Creely, A. E. Martin and C. M. Conrad, *Text. Res. J.*, 1959, **29**, 786–794.
